# Kidney Ischemia-Reperfusion Elicits Acute Liver Injury and Inflammatory Response

**DOI:** 10.3389/fmed.2020.00201

**Published:** 2020-06-02

**Authors:** Yue Shang, Susara Madduma Hewage, Charith U. B. Wijerathne, Yaw L. Siow, Cara K. Isaak, Karmin O

**Affiliations:** ^1^St. Boniface Hospital Research Centre, Winnipeg, MB, Canada; ^2^Department of Animal Science, University of Manitoba, Winnipeg, MB, Canada; ^3^Department of Physiology and Pathophysiology, University of Manitoba, Winnipeg, MB, Canada; ^4^Agriculture and Agri Food Canada, St. Boniface Hospital Research Centre, Winnipeg, MB, Canada

**Keywords:** kidney, liver, ischemia-reperfusion, inflammation, acute kidney injury

## Abstract

Ischemia-reperfusion (IR) is a common risk factor that causes acute kidney injury (AKI). AKI is associated with dysfunction of other organs also known as distant organ injury. The liver function is often compromised in patients with AKI and in animal models. However, the underlying mechanisms are not fully understood. Inflammatory response plays an important role in IR-induced tissue injury. Although increased proinflammatory cytokines have been detected in the kidney and the distant organs after renal IR, their original sources remain uncertain. In the present study, we investigated the acute effect of renal IR on hepatic inflammatory cytokine expression and the mechanism involved. Sprague-Dawley rats that were subjected to renal IR (ischemia for 45 min followed by reperfusion for 1 h or 6 h) had increased plasma levels of creatinine, urea, and transaminases, indicating kidney and liver injuries. There was a significant increase in the expression of proinflammatory cytokine mRNA (MCP-1, TNF-α, IL-6) in the kidney and liver in rats with renal IR. This was accompanied by a significant increase in proinflammatory cytokine protein levels in the plasma, kidney, and liver. Activation of a nuclear transcription factor kappa B (NF-κB) was detected in the liver after renal IR. The inflammatory foci and an increased myeloperoxidase (MPO) activity were detected in the liver after renal IR, indicating hepatic inflammatory response and leukocyte infiltration. These results suggest that renal IR can directly activate NF-κB and induce acute production of proinflammatory cytokines in the liver. Renal IR-induced hepatic inflammatory response may contribute to impaired liver function and systemic inflammation.

## Introduction

Acute kidney injury (AKI) is characterized by a rapid decline in kidney function over a short period of time and is associated with a high mortality. AKI often leads to multiple organ dysfunctions known as distant organ injury (1–5). Kidney ischemia-reperfusion (IR) is one of the most common causes for AKI (6–8). It occurs in clinical situations such as kidney transplantation, cardiac surgery, sepsis, and in critically ill patients. Despite the advancement in renal replacement therapy, the mortality in patients with AKI that is complicated by multiple-organ dysfunction remains high worldwide (estimated to be 50%). In experimental animals, renal IR is shown to cause distant organ injury in the heart, lung, brain, intestine, and liver (1, 9–15). Clinical studies have shown that AKI patients with multiple organ dysfunctions have worse prognosis than those who have AKI alone (16, 17). Although the remote effects of AKI have been noted for a long time, the precise mechanisms responsible for pathological changes in the distant organs are not well-understood.

Liver function is often impaired in patients with AKI and in animal models with renal IR or nephrectomy (1, 9, 15, 18). It appears that AKI patients with a complication of liver dysfunction have poorer clinical outcomes (16, 17, 19, 20). Oxidative stress, systemic inflammatory response and increased leukocyte trafficking have been implicated in AKI associated distant organ injury. In experimental animals, AKI-induced liver injury manifests with increased oxidative stress, hepatocyte necrosis, elevated cytokines, and leukocyte infiltration (3, 15, 16, 21). Liver plays a central role in metabolism, redox balance, immune regulation, and detoxification. Our recent study has shown that renal IR causes liver injury with reduced hepatic production of glutathione, a major endogenous antioxidant. We have identified that renal IR directly inhibits hepatic glutathione production through downregulation of Nrf-2 mediated glutathione biosynthesis pathway, leading to oxidative stress in rats (15). Renal IR-induced redox imbalance in the liver may contribute to local and systemic oxidative stress (15, 21).

Inflammatory response plays a critical role in IR-induced tissue injury (22). Upon renal IR, proinflammatory cytokines generated in the kidney are considered as major contributors to local and systemic inflammation (13, 23–26). Nuclear factor-kappa B (NF-κB) is one of the key transcription factors that regulates the expression of proinflammatory cytokines and immune response (26, 27). Our previous study showed that IR stimulated chemokine MCP-1 expression through the activation of NF-κB in the kidney (26, 28). Although increased levels of cytokine proteins in the liver were detected in mice 5 h after renal IR (16), in rats 6 h and 24 h after renal IR (21) and in pigs 48 h after renal IR (29), the origin(s) of increased cytokines in the liver after renal IR were not well-identified. It was suggested that the remote effect of renal IR might be initiated by impaired renal clearance or increased influx of cytokines derived from the kidney or from increased mononuclear phagocyte production (24). However, it is unclear whether renal IR can acutely elicit distant organ inflammatory response through directly triggering NF-κB activation and cytokine expression in the liver. Further research is required to identify the molecular mechanisms underlying renal IR-induced distant organ inflammatory response. In the present study, we investigated the early impact of renal IR on liver function and inflammatory response as well as the potential mechanism involved.

## Materials and Methods

### Animal Model

Renal IR was induced in rats as described in our previous studies (15, 28, 30, 31). In brief, Sprague–Dawley rats (250–300 g, male, Central Animal Care Services, University of Manitoba, Winnipeg, MB, Canada) were anesthetized through inhalation of 3% isoflurane/oxygen gas prior to surgery. Renal ischemia was induced by clamping the left kidney pedicle for 45 min. At the end of ischemia, the clamp was removed to allow reperfusion in the left kidney with right nephrectomy. Rats were sacrificed at 1 or 6 h after reperfusion. As a control (sham-operated), rats were subjected to the same surgical procedure without inducing renal ischemia and were sacrificed at the corresponding time points. Blood was collected and centrifuged at 3,000 *g* for 20 min for plasma preparation. All procedures were performed in accordance with the Guide to the Care and Use of Experimental Animals published by the Canadian Council on Animal Care and approved by the University of Manitoba Protocol Management and Review Committee.

### Biochemical Analysis

Plasma creatinine, urea, alanine aminotransferase (ALT), and aspartate aminotransferase (AST) were measured by using the Cobas C111 Analyzer (Roche, Risch-Rotkreuz, Switzerland). Cytokines in the plasma, kidney, and liver were measured by using the electrochemiluminescent sandwich ELISA array from Meso Scale Discovery (Rockville, MD, USA). Liver myeloperoxidase (MPO) activity was measured by using a fluorometric assay with a commercial MPO Activity Assay Kit (ab111749, Abcam Inc., Toronto, ON, Canada).

### Real-Time Polymerase Chain Reaction (PCR) Analysis

Total RNAs were isolated from the kidney and liver with Trizol reagent (Invitrogen, Carlsbad, CA, USA). The mRNA of MCP-1, TNF-α, and IL-6 was determined by a real-time polymerase chain reaction (PCR) analysis using the iQ5 real-time PCR detection system (Bio-Rad, Mississauga, ON, Canada) and normalized with β-actin (23, 31, 32). The primers (Thermo Fisher Scientific, Waltham, MA, USA) used for rat mRNA measurement were: MCP-1 (119 bp), 5′- CAGAAACCAGCCAACTCTCA-3′ (forward) and 5′- AGACAGCACGTGGATGCTAC-3′ (reverse) (GeneBank accession number NM_031530), TNF-α (215 bp), 5′- CCCAGACCCTCACACTCAGAT-3′ (forward) and 5′- TTGTCCCTTGAAGAGAACCTG-3′ (reverse) (GenBank, accession number NM_012675), IL-6 (161 bp), 5′- CCGGAGAGGAGACTTCACAG-3′ (forward) and 5′-ACAGTGCATCATCGCTGTTC-3′ (reverse) (GenBank accession number NM_012589) and β-actin (198 bp), 5′- ACAACCTTCTTGCAGCTCCTC-3′ (forward) and 5′- GACCCATACCCACCA TCACA-3′ (reverse) (GenBank accession number NM_031144).

### Electrophoretic Mobility Shift Assay (EMSA)

The binding activity of NF-κB with DNA was measured by electrophoretic mobility shift assay (EMSA) (Thermo Fisher Scientific, Waltham, MA, USA). In brief, nuclear proteins were prepared from the liver after renal IR or sham-operation as described in our previous studies (31). Nuclear proteins (2 μg) were incubated with biotin-labeled oligonucleotides containing a consensus sequence specific for the NF-κB/DNA binding site (5′-AGTTGAGGGGACTTTCCCAGGC-3′) (Promega, Madison, WI, USA). To confirm an equal loading of proteins for each sample, nuclear histone H3 protein was measured by Western immunoblotting analysis. Liver nuclear proteins (10 μg) were separated by electrophoresis in a 12% SDS-polyacrylamide gel. Proteins in the gel were transferred to a nitrocellulose membrane that was first probed with anti-histone H3 polyclonal antibodies (1:1,000, SC-10809, Dallas, TX, USA) followed by HRP-conjugated anti-rabbit IgG secondary antibodies (1:1,000, #7074, New England Biolabs, Ipswich, MA, USA). The protein bands were visualized by using Luminata Crescendo chemiluminescent HRP detection reagent (Millipore, Burlington, MA, USA) and quantified with the Quantity One 1-D Analysis Software (Bio-Rad).

### Histological Examination

For histological examination, a portion of the kidney or liver was immersion fixed in 10% neutral-buffered formalin followed by embedding in paraffin. The paraffin-embedded cross sections (5 μm) were prepared and stained with hematoxylin and eosin (H&E) to examine histological changes in the kidney and the liver as described in our previous studies (28). The images were captured by using Olympus BX43 Upright Light Microscope with an Olympus QColor3 digital camera (Olympus Corporation, Tokyo, Japan) and analyzed using Image-Pro plus 7.0 (Media Cybernetics, Bethesda, MD, USA).

### Statistical Analysis

Results were analyzed using a two-tailed Student's *t*-test. *P*-value < 0.05 were considered statistically significant.

## Results

### Renal Ischemia-Reperfusion Impaired Kidney and Liver Function

**Figure 1:**
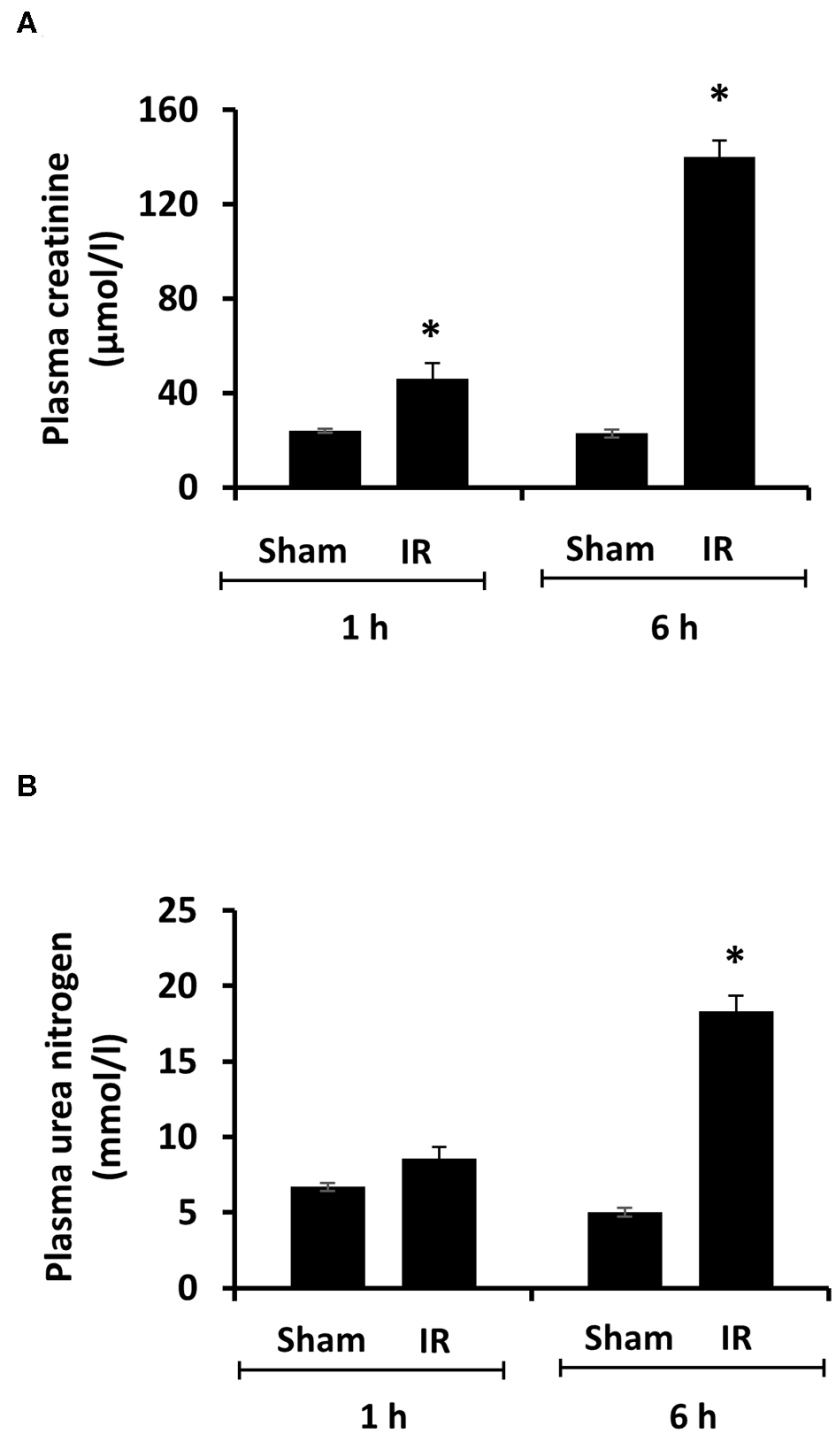
Effect of kidney ischemia-reperfusion on kidney function. The left kidney of rats was subjected to 45 min ischemia followed by 1 hour (1 h) or 6 hours (6 h) of reperfusion (IR). As a control, rats were subjected to a sham-operation without inducing ischemia (Sham). Plasma creatinine **(A)** and urea nitrogen **(B)** were measured. Results are expressed as mean ± SE (*n* = 5 for each group). *p < 0.05 when compared with the value obtained from the sham-operated group.

**Figure 2:**
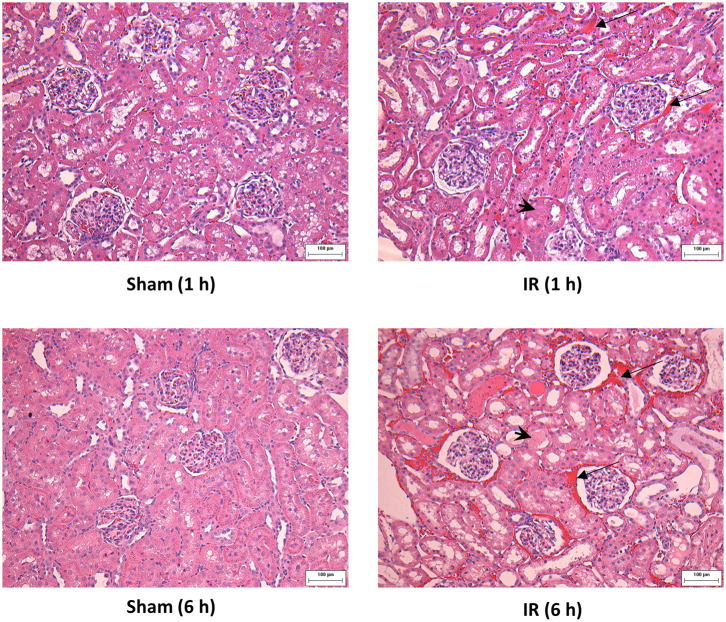
Effect of kidney ischemia-reperfusion on histological change in the kidney. The left kidney of rats was subjected to 45 min ischemia followed by 1 hour (1 h) or 6 hours (6 h) of reperfusion (IR). As a control, rats were subjected to a sham-operation without inducing ischemia (Sham). The histological structure of kidney was examined by hematoxylin and eosin (H&E) staining and analyzed at ×200 magnification. Kidneys of the IR group showed tubular necrosis (arrowheads), interstitial congestion of red blood cells (arrow), and glomerulus enlargement compared with the Sham group (scale bar = 100 μm).

**Figure 3:**
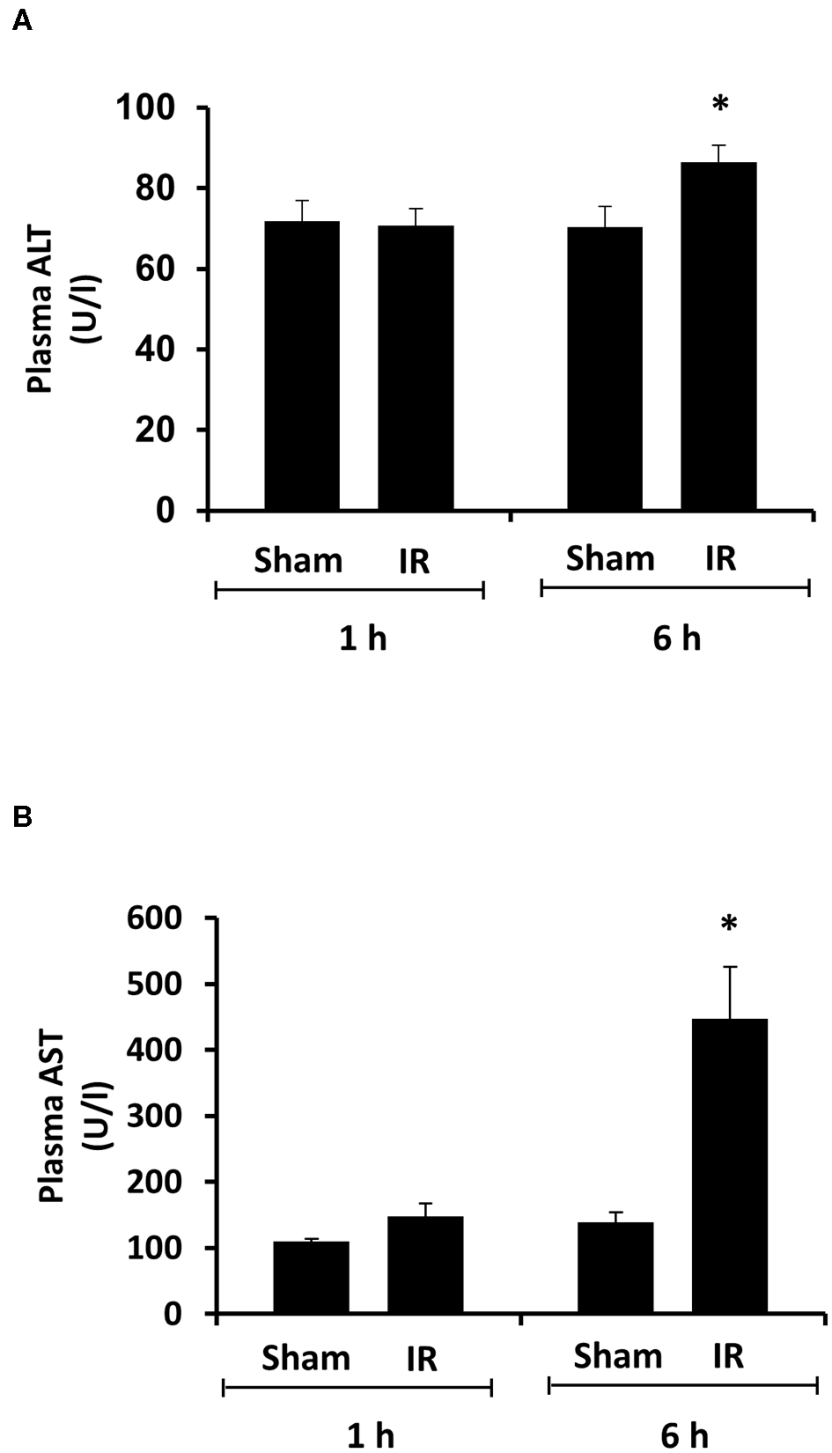
Effect of kidney ischemia-reperfusion on liver function. The left kidney of rats was subjected to 45 min ischemia followed by 1 hour (1 h) or 6 hours (6 h) of reperfusion (IR). As a control, rats were subjected to a sham-operation without inducing ischemia (Sham). Plasma alanine transaminase (ALT) **(A)** and aspartate aminotransferase (AST) **(B)** were measured. Results are expressed as mean ± SE (*n* = 5 for each group). *p < 0.05 when compared with the value obtained from the sham-operated group.

Renal ischemia for 45 min followed by reperfusion for 1 or 6 h caused a significant elevation of plasma creatinine and urea levels (Figure 1), indicating that IR impaired kidney function. IR also caused histological changes in the kidney. The H&E staining showed tubular necrosis, glomerulus enlargement and interstitial congestion of red blood cells in the kidneys of rats subjected to renal IR (Figure 2). There was a significant increase in plasma ALT and AST levels in rats at 6 h after renal IR as compared to sham-operated rats (Figure 3), indicating liver injury. These results suggested that renal IR not only caused kidney injury but also impaired liver function.

### Elevation of Proinflammatory Cytokine Levels in the Kidney, Liver, and Plasma

**Figure 4:**
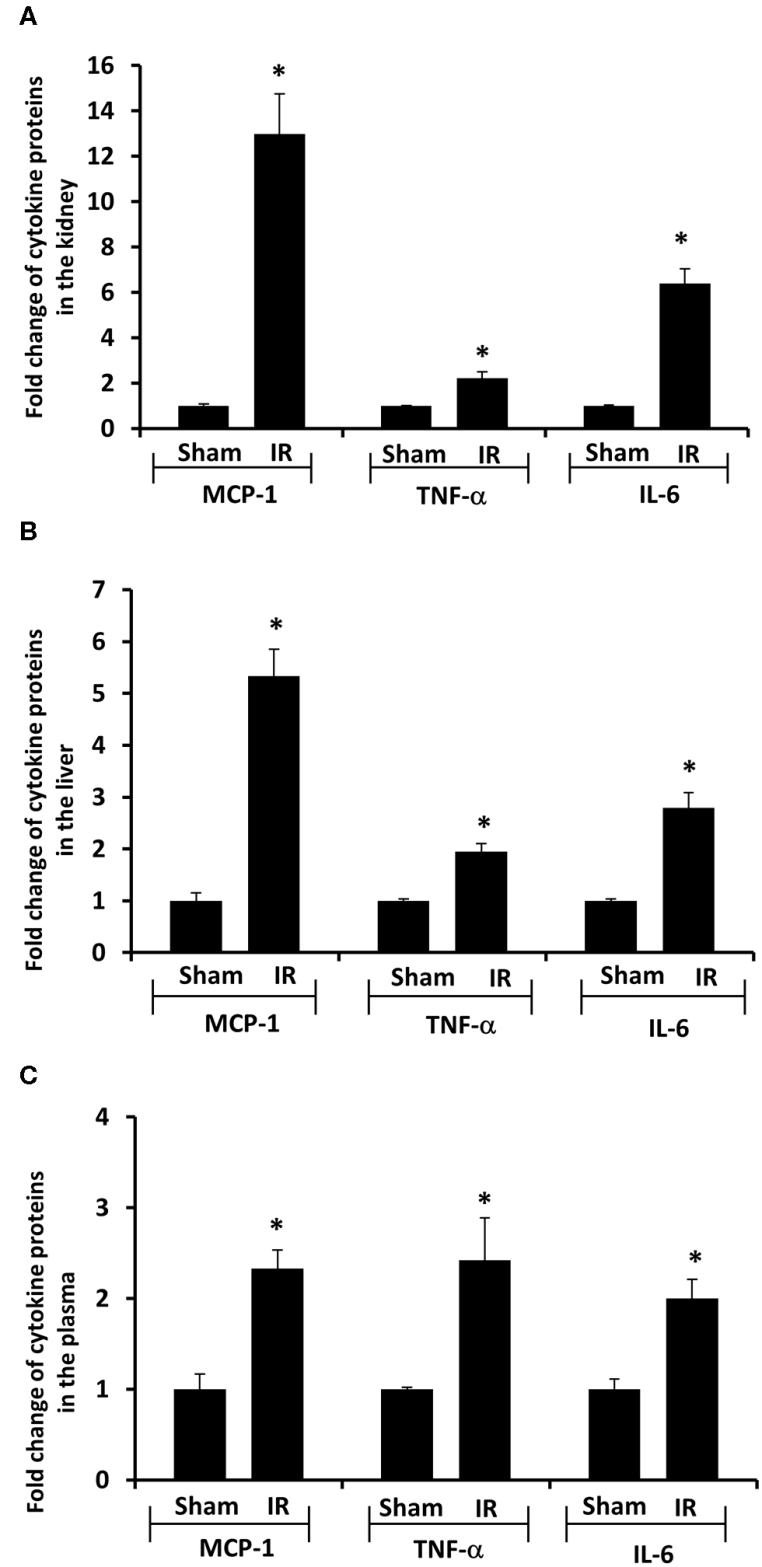
Effect of kidney ischemia-reperfusion on cytokine protein levels in the kidney, liver, and plasma. The left kidney of rats was subjected to 45 min ischemia followed by 6 hours (6 h) of reperfusion (IR). As a control, rats were subjected to a sham-operation without inducing ischemia (Sham). The protein expressions of MCP-1, TNF-α, and IL-6 were measured in the kidney **(A)**, liver **(B)**, and plasma **(C)** by using ELISA. For reference, the cytokine levels in the sham group were MCP-1 (kidney 3.9 ng/g tissue, liver 100 pg/g tissue, plasma 13.63 ng/ml), TNF-α (kidney 200 ng/g tissue, liver 41 pg/g tissue, plasma 1.2 pg/ml), IL-6 (kidney 3.2 ng/g tissue, liver 1,880 pg/g tissue, plasma 1.8 pg/ml). Results are expressed as fold change to the Sham (*n* = 5 for each group). *p < 0.05 when compared with the value obtained from the sham-operated group.

Next, we measured proinflammatory cytokines in the plasma, kidney, and liver. Renal IR resulted in a significant increase in TNF-α, IL-6, and MCP-1 protein levels in the kidney, liver and plasma (Figure 4). These results suggested that renal IR not only increased local inflammatory response but also elevated proinflammatory cytokine levels in a distant organ (liver) and in the circulation.

### Renal Ischemia-Reperfusion Increased the Expression of Proinflammatory Cytokine Expression in The Kidney and Liver

**Figure 5:**
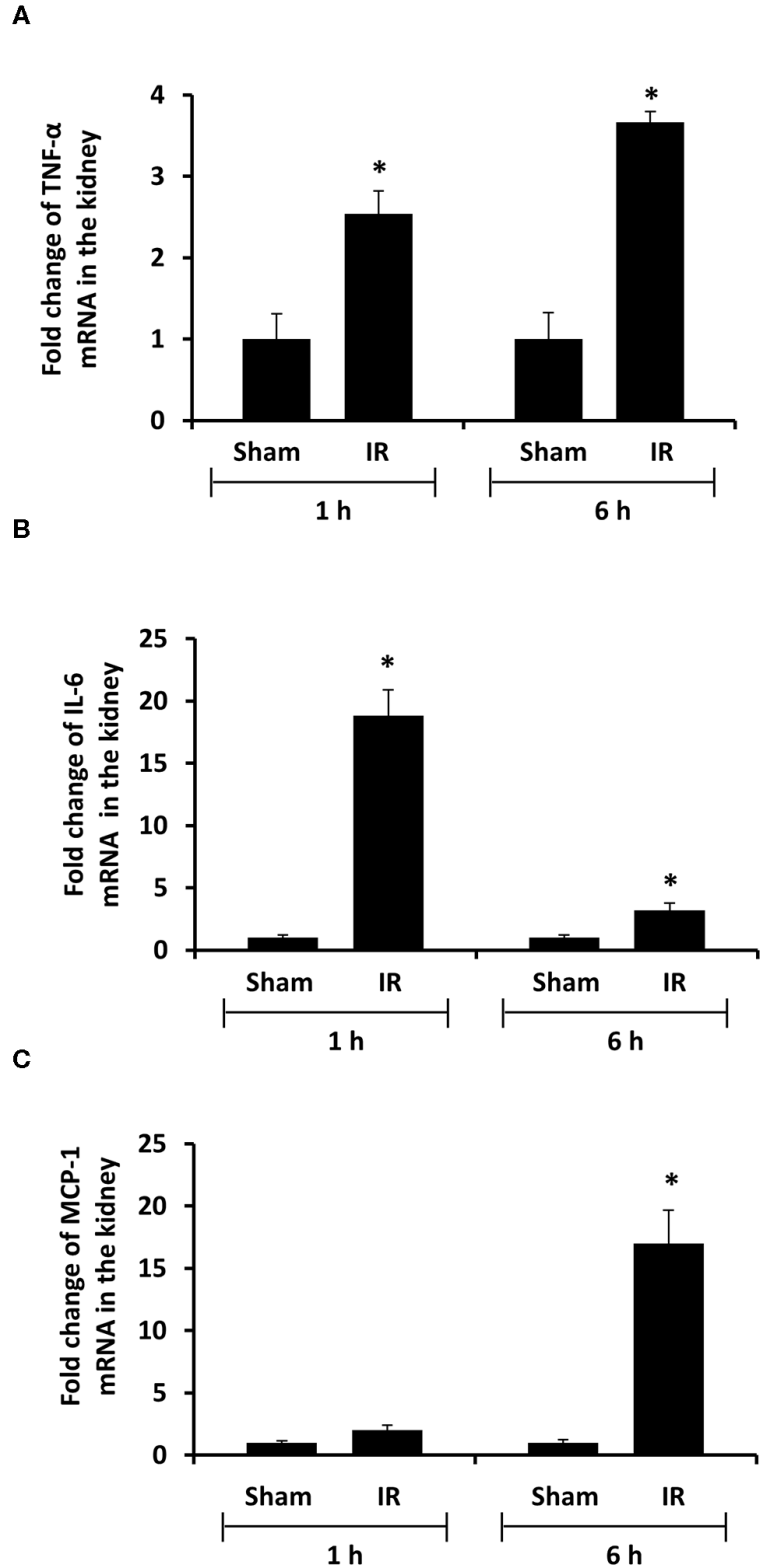
Effect of kidney ischemia-reperfusion on cytokine mRNA expression in the kidney. The left kidney of rats was subjected to 45 min ischemia followed by 1 hour (1 h) or 6 hours (6 h) of reperfusion (IR). As a control, rats were subjected to a sham-operation without inducing ischemia (Sham). The mRNA expressions of TNF-α **(A)**, IL-6 **(B)**, and MCP-1 **(C)** were determined in the kidney by a real-time PCR analysis. Results are expressed as fold change to the Sham (*n* = 5 for each group). *p < 0.05 when compared with the value obtained from the sham-operated group.

**Figure 6:**
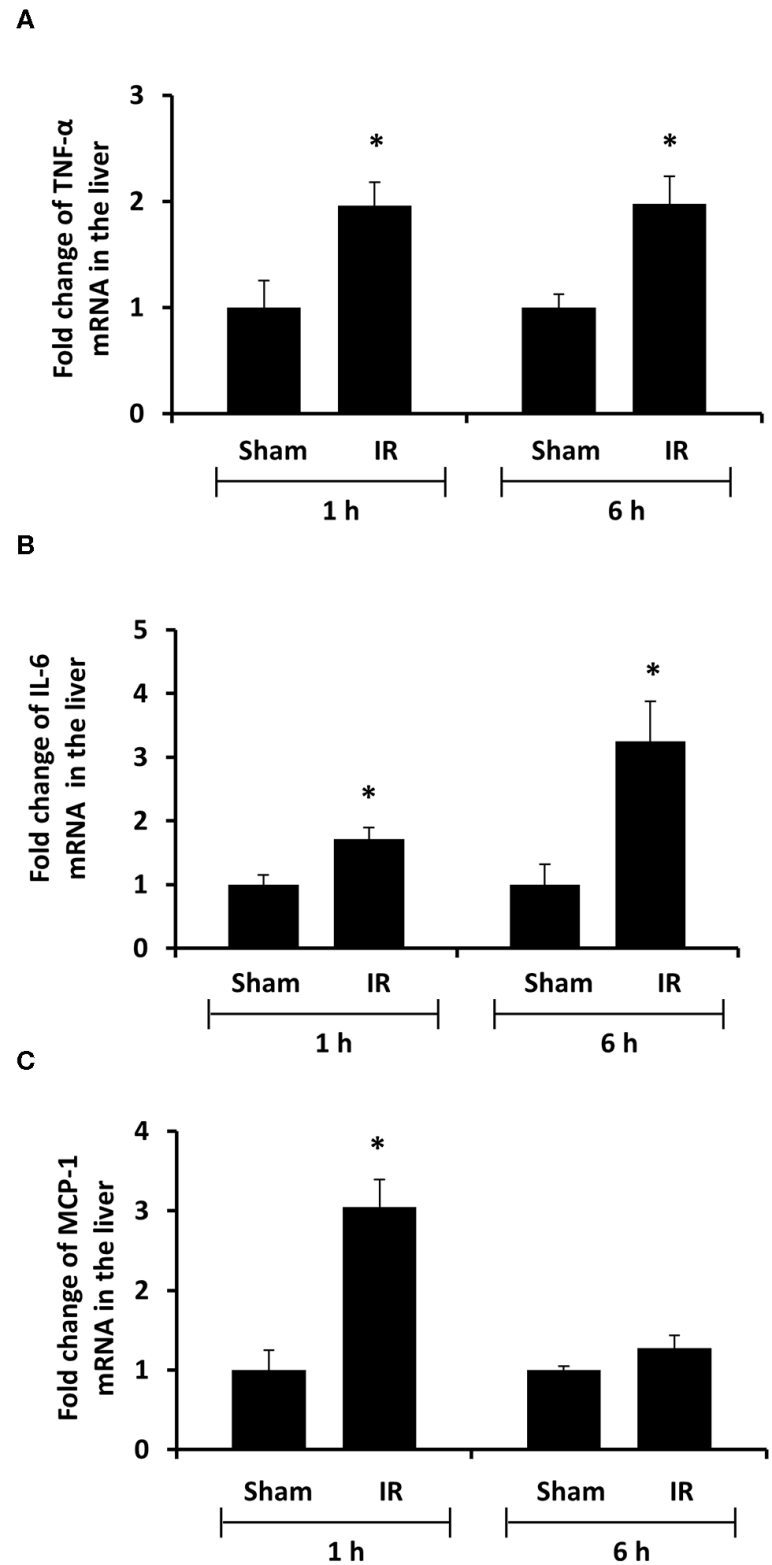
Effect of kidney ischemia-reperfusion on cytokine mRNA expression in the liver. The left kidney of rats was subjected to 45 min ischemia followed by 1 hour (1 h) or 6 hours (6 h) of reperfusion (IR). As a control, rats were subjected to a sham-operation without inducing ischemia (Sham). The mRNA expression of TNF-α **(A)**, IL-6 **(B)**, and MCP-1 **(C)** was determined in the liver by a real-time PCR analysis. Results are expressed as fold change to the Sham (*n* = 5 for each group). *p < 0.05 when compared with the value obtained from the sham-operated group.

To investigate the source of increased proinflammatory cytokines in the local and distant organs, we measured proinflammatory cytokine gene expression in the kidney and liver. There was a significant increase in TNF-α, IL-6, and MCP-1 mRNA in both kidney and liver tissues in rats that were subjected to renal IR (Figures 5, 6). A significant elevation of TNF-α and IL-6 mRNA was detected in the kidney (Figures 5A,B) and liver (Figures 6A,B) at 1 h or 6 h after renal IR. However, an elevation of MCP-1 mRNA expression was detected in the kidney at 6 h after renal IR (Figure 5C). Interestingly, an elevation of MCP-1 mRNA expression was detected in the liver at 1 h after IR (Figure 6C). Taken together, these results suggested that renal IR not only caused local inflammatory response but also stimulated the expression of proinflammatory cytokines in the distant organ(s), which might contribute, in part, to an elevation of these cytokines in the circulation.

### Renal Ischemia-Reperfusion Activated Transcriptional Factor NF-κB in the Liver

**Figure 7:**
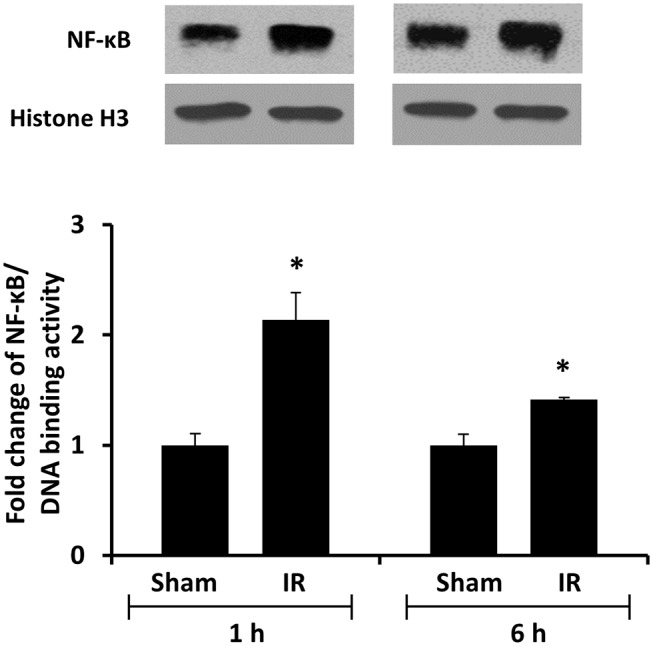
The DNA binding activity of NF-κB in the liver. The left kidney of rats was subjected to 45 min ischemia followed by 1 hour (1 h) or 6 hours (6 h) of reperfusion (IR). As a control, rats were subjected to a sham-operation without inducing ischemia (Sham). Liver nuclear proteins were prepared. The DNA binding activity of NF-κB in the liver was determined by EMSA. Histone H3 was determined by Western immunoblotting analysis and used as an internal control. Results are expressed as fold change to the Sham (*n* = 5 for each group). *p < 0.05 when compared with the value obtained from the sham-operated group.

**Figure 8:**
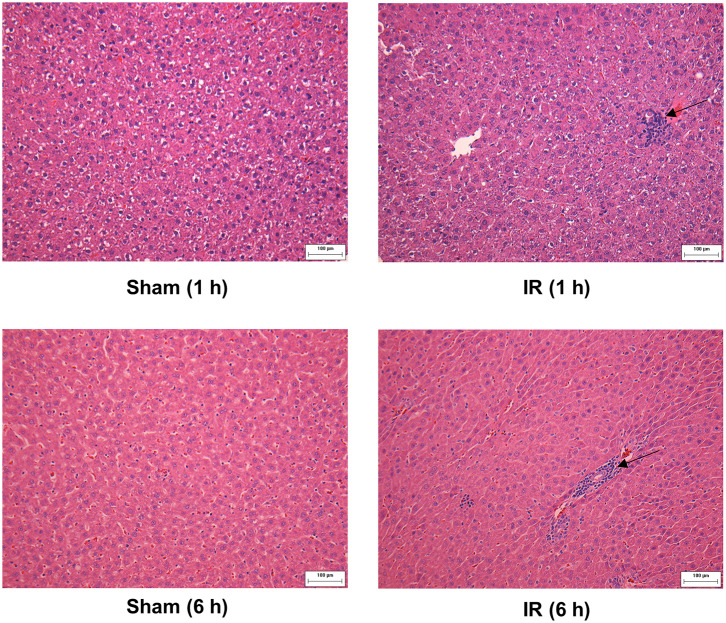
Effect of kidney ischemia-reperfusion on pathohistological change of the liver. The left kidney of rats was subjected to 45 min ischemia followed by 1 hour (1 h) or 6 hours (6 h) of reperfusion (IR). As a control, rats were subjected to a sham-operation without inducing ischemia (Sham). The histological structure of liver was examined by hematoxylin and eosin (H&E) staining (magnification ×200). Arrows point to inflammatory foci (scale bar = 100 μm).

**Figure 9:**
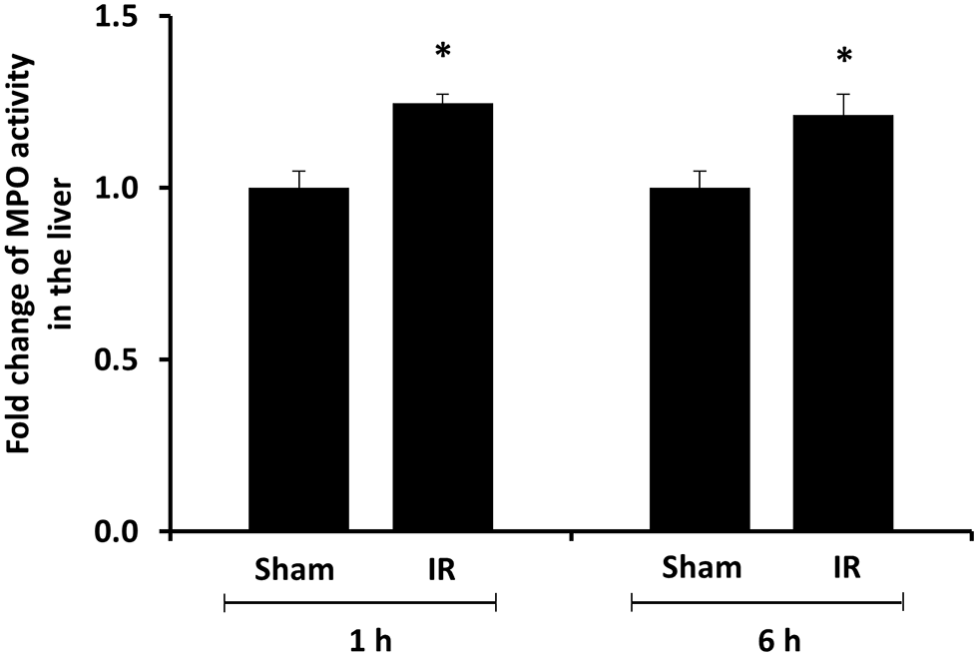
Effect of kidney ischemia-reperfusion on myeloperoxidase activity in the liver. The left kidney of rats was subjected to 45 min ischemia followed by 1 hour (1 h) and 6 hours (6 h) of reperfusion (IR). As a control, rats were subjected to a sham-operation without inducing ischemia (Sham). The myeloperoxidase (MPO) activity were determined in the liver. Results are expressed as fold change to the Sham. Results are expressed as mean ± SE (*n* = 4–5 for each group). *p < 0.05 when compared with the value obtained from the sham-operated group.

We previously observed that renal IR caused an activation of NF-κB, a main transcription factor for cytokine expression, in the kidney (26). To investigate whether renal IR also activated NF-κB in a distant organ, liver nuclear proteins were prepared and the activation of NF-κB was examined by EMSA. There was a significant increase in the NF-κB/DNA binding activity in the liver of rats at 1 h or 6 h after renal IR (Figure 7). The liver morphology was examined by H&E staining. There was deposition of inflammatory foci (characterized by dense aggregates of cells) in the liver of rats after renal IR (Figure 8). The MPO activity was measured to determine the presence of neutrophils in the liver. The hepatic MPO activity was significantly increased as early as 1 h after renal IR and remained elevated at 6 h after renal IR (Figure 9).

## Discussion

In the present study, rats that were subjected to renal IR developed kidney and liver injuries as manifested by increased plasma creatinine, urea, and transaminases (ALT, AST) levels along with histological changes. Increased proinflammatory cytokine mRNA expression was detected in the kidney and liver shortly after the onset of renal IR. In correspondence, the levels of inflammatory cytokine proteins (MCP-1, TNF-α, IL-6) were significantly elevated in the kidney, liver and plasma. These results suggest that renal IR can quickly stimulate inflammatory cytokine production locally as well as in distant organs such as in the liver, which augments systemic inflammation and may, in turn, exacerbate kidney injury.

Previous studies conducted by our laboratory and others showed an elevation of proinflammatory cytokine expression in the kidney upon IR (16, 23, 24, 26). Increased proinflammatory cytokine expression is considered as one of the important mechanisms for IR-induced tissue injuries. Increased levels of cytokine proteins were detected in the circulation and distant organs in mice and rats with IR or nephrectomy-induced AKI (16, 33). However, the origin(s) of elevated proinflammatory cytokines have not been well-defined. It has been suggested that an increased production and a decreased clearance by the kidneys upon IR may lead to elevated cytokine levels in the circulation and the distant organs (14).

The present study revealed a significant increase in proinflammatory cytokine levels in the kidney, liver, and plasma at 6 h after renal IR. Although it was plausible that proinflammatory cytokines produced in originally injured organ, namely, kidney could lead to systemic and distant organ inflammation, several lines of evidence indicated that renal IR could also stimulate the production of cytokines in a distant organ. First, activation of NF-κB was detected in the liver of rats at the early time points 1 h and 6 h after renal IR. NF-κB is a transcription factor that plays an important role in the inflammatory response by up-regulating cytokine expression. Multiple pathways have been proposed in the activation of NF-κB. We previously reported that renal IR led to a significant elevation of plasma homocysteine (Hcy) levels (15, 23, 30, 34). Hcy at an elevated level, which is also known as hyperhomocysteinemia, can elicit inflammatory response through activation of transcription factors including NF-κB (35–37). It was plausible that Hcy at elevated levels might serve as one of mediators that contributed to NF-κB activation in the distance organs such as liver during renal IR. Acute activation of NF-κB in response to renal IR could lead to a rapid increase in inflammatory cytokine expression in the liver. Secondly, an elevation of TNF-α, IL-6, and MCP-1 mRNA expression was detected in the liver as early as 1 h after the onset of renal IR, suggesting that an acute up-regulation of proinflammatory cytokine production occurred in a distant organ (liver). This, in turn, might contribute to increased cytokine levels in the circulation. MCP-1 is a potent chemotactic protein that stimulates monocyte/neutrophil infiltration into tissues. We previously reported that there was a significant increase in MCP-1 expression in the kidney at 2 h after renal IR (26). The expression of MCP-1 in the kidney remained elevated 24 h after renal IR (23, 26). Up-regulation of MCP-1 expression by IR might be tissue-specific and time-dependent. In the present study, a significant elevation of MCP-1 mRNA expression was detected in the liver but not in the kidney at 1 h after renal IR. However, a stimulation of MCP-1 expression in the liver was rather transient when compared to its expression in the kidney upon renal IR. Renal IR also stimulated the expression of other proinflammatory cytokines. An elevation of TNF-α and IL-6 mRNA expression was detected in the kidney and liver at 1 or 6 h after renal IR. Furthermore, inflammatory foci were detected in the liver of rats that were subjected to renal IR. This was accompanied by an increased MPO enzymatic activity in the liver tissue. The MPO is a peroxidative enzyme that is abundantly expressed in neutrophil granulocytes. An elevation of hepatic MPO activity indicated neutrophil accumulation in the liver after renal IR. However, the involvement of other inflammatory cells in distant organ injury remains to be investigated. Taken together, these results suggested that increase proinflammatory cytokine production in the kidney and the distant organ(s) could lead to an elevation of cytokine levels in the circulation, which, in turn, might contribute to systemic inflammation during AKI. Future studies are warranted to investigate whether early intervention can alleviate renal IR-induced local and distant organ inflammatory response.

In conclusion, renal IR not only causes kidney injury but also results in liver dysfunction. Inflammation is a hallmark of IR-induced kidney and distant organ injury, which can augment the already high morbidity and mortality in patients with AKI. Although increased proinflammatory cytokine production in the kidney is regarded as one of the important mechanisms leading to systemic and distant organ injury, our results clearly demonstrate that renal IR can directly induce acute production of proinflammatory cytokines in the liver. This, in turn, may contribute to systemic inflammatory response and exacerbate kidney injury. Activation of NF-κB may be involved in renal IR-induced inflammatory cytokine production in the liver. Better understanding of the local and distant organ responses to AKI will help identify new therapeutic targets and ultimately improve the clinical outcomes.

## Data Availability Statement

All datasets generated for this study are included in the article/supplementary material.

## Ethics Statement

The animal study was reviewed and approved by University of Manitoba Protocol Management and Review Committee.

## Author Contributions

KO and YLS conceived and designed research and drafted the manuscript. YS, CW, SM, and CI performed the experiments and analyzed the data. YS, CW, and SM prepared the figures. CW, SM, YS, CI, YLS, and KO edited and revised the manuscript.

## Conflict of Interest

The authors declare that the research was conducted in the absence of any commercial or financial relationships that could be construed as a potential conflict of interest.
